# Automated generation of consistent annual maximum NDVI on coal bases with a new algorithm

**DOI:** 10.1038/s41597-024-03543-2

**Published:** 2024-06-26

**Authors:** Jun Li, Tingting Qin, Chengye Zhang, Yicong Zhang, Yaping Zhang, Haitao Shi, Yihao Yang

**Affiliations:** 1 College of Geoscience and Surveying Engineering, China University of Mining and Technology, Beijing, 100083 Beijing China; 2 State Key Laboratory of Coal Fine Exploration and Intelligent Development, China University of Mining and Technology, Beijing, 100083 Beijing China

**Keywords:** Environmental impact, Restoration ecology, Energy and behaviour

## Abstract

Coal is one of the most important fossil energy sources and is ensuring global energy security. Annual maximum NDVI (Normalized Difference Vegetation Index) data is an important indicator for the research in balancing coal mining and vegetation conservation. However, the existing annual maximum NDVI data displayed lower values with temporally inconsistent and a noticeable mosaic line. Here we propose an algorithm for automatically generating the annual maximum NDVI of China’s coal bases in Google Earth Engine called: Auto-NDVI_cb_. The accuracy of the Auto-NDVI_cb_ algorithm has been verified with an average RMSE of 0.087 for the 14 coal bases from 2013 to 2022. Based on the proposed Auto-NDVI_cb_ algorithm, an annual maximum NDVI dataset for all 14 coal bases in China from 2013 to 2022 was publicly released. This dataset can be fast and automatically updated online. Hence, the public dataset will continuously serve to monitor the vegetation change induced by coal mining, exploring the mechanism of vegetation degradation, and providing scientific data for developing vegetation protection policies in coal mines.

## Background & Summary

Energy and the environment are at the centre of the world’s current concerns. As one of the most important fossil energy sources, coal has been playing a crucial role in ensuring global energy security. However, coal exploitation has led to a range of ecological and environmental challenges, in particular, the degradation of vegetation, which is getting more and more attention from the major coal-producing countries of the world, such as America, Australia, and China^1–4^.

Normalized Difference Vegetation Index (NDVI) is a popular and effective indicator for characterizing the growth and coverage of vegetation. Since it was proposed in the 1970s^5^, numerous researchers have successfully used NDVI to characterize vegetation status in terms of spatial and temporal change^6–11^. For example, Gao *et al*.^8^ used NDVI as an indicator for statistical regression to analyse vegetation changes at different elevations over the past 30 years. Yang *et al*.^12,13^ used the NDVI time series and the shape-based clustering method for monitoring vegetation change in a surface coal mining area. Li *et al*.^14^ used NDVI data to investigate the vegetation cover to reflect the disturbance and restoration of the opencast mining areas. Therefore, high-quality NDVI data are urgently needed to monitor vegetation changes in coal bases. It can not only improve the knowledge of ecological changes in the mining area but also guide and optimize production and ecological restoration activities in the mining area. Due to mining and restoration activities, time-series change of vegetation at the object-scale in mining areas is usually temporally abrupt. Moreover, the inter-annual variation in phenology also influences the NDVI in a similar season every year. The annual maximum NDVI data better reflects the real growth of the vegetation.

There are two main methods for acquiring NDVI data. The first method is to retrieve NDVI data directly from publicly available NDVI products. The second method is to perform the own band math to generate NDVI by the users.

1. Using publicly available NDVI products. Some NDVI products based on different satellite sensors have been produced by various institutions or experts over the decades. The primary NDVI products are listed in Table 1, involved with satellites/sensors such as the Advanced Very High-Resolution Radiometer (AVHRR)^15–17^, SPOT/VEGETATION, Moderate Resolution Imaging Spectroradiometer (MODIS)^18,19^, Visible Infrared Imaging Radiometer Suite (VIIRS)^20,21^, GF series^22^, Landsat series^23^, and Sentinel series^24^. The spatial resolution of existing NDVI products varies from 10 km (e.g., GIMMS NDVI) to 1 km-250 m (e.g., MODIS NDVI), 30 m (Landsat NDVI), and even 10 m (Sentinel-2 NDVI). Products such as “GIMMS NDVI From AVHRR Sensors”, “NOAA CDR AVHRR NDVI”, and “MODIS Terra Daily NDVI” offer high temporal resolution but low spatial resolution, making them unsuitable for small-scale studies. Low-resolution NDVI images may not capture fine vegetation features within mining areas, such as small vegetation cover or unevenly distributed vegetation. Additionally, the presence of multiple surface cover types around the coal base, including bare soil, ore stockpiles, vegetation, etc., can lead to the mixing of these diverse surfaces in a single image element. The accuracy of vegetation analysis results may be compromised. Conversely, “China 10 m Year-by-Year NDVI Maximum Dataset (2016–2021)” and “MuSyQ GF-series 16 m/10-day NDVI Vegetation Index Dataset (2018–2020)”^25^ have a higher spatial resolution, but only provide data for recent several years, which cannot satisfy the long-time series research. Landsat satellites combine the advantages of both temporal and spatial resolution.

**Table 1:** Publicly Available NDVI datasets.

Satellites/Sensors	Datasets	Spatial resolution	Period/ temporal resolution	Available Link
AVHRR	NOAA CDR of AVHRR NDVI^52^	0.05 degree	1981–2013/10-Day	https://www.ncei.noaa.gov/metadata/geoportal/rest/metadata/item/gov.noaa.ncdc:C01558/html#Description1
	GIMMS NDVI From AVHRR Sensors (3rd Generation)^53^	1/12 degree	1981–2015/8-Day	https://climatedataguide.ucar.edu/climate-data/ndvi-normalized-difference-vegetation-index-3rd-generation-nasagfsc-gimms
PROBA	PROBA-V L3 TOC NDVI PRODUCTS^54,55^	1 km/300 m/100 m	2013–2020/1, 5 or 10-Day	https://proba-v.vgt.vito.be/en/product-types/c2/level-3toc
MODIS	MODIS Combined 16-Day NDVI^56,57^	250 m/500 m/1 km	2000-Present/16-Day	https://modis.gsfc.nasa.gov/data/dataprod/mod13.php
	MODIS/Terra Vegetation Indices Monthly L3 Global 1 km SIN Grid^58^	1 km	2001-Present/Monthly	https://ladsweb.modaps.eosdis.nasa.gov/missions-and-measurements/products/MOD13A3
VIIRS	VNP13A1: VIIRS Vegetation Indices 16-Day 500m^59^	500 m	2012-Present/16-Day	https://lpdaac.usgs.gov/products/vnp13a1v001/#nav-heading
Landsat	China annual maximum NDVI dataset^60^	30 m	2000–2020/Yearly	http://www.nesdc.org.cn/
	China annual maximum NDVI dataset^31^	30 m	1986–2021/Yearly	https://www.resdc.cn/doi/
	8-Day NDVI Composite^48^	30 m	1986–2021/8-Day	https://developers.google.com/earth-engine/datasets
	32-Day NDVI Composite^48^	30 m	1986–2021/32-Day	https://developers.google.com/earth-engine/datasets
	Annual NDVI Composite^48^	30 m	1986–2021/Yearly	https://developers.google.com/earth-engine/datasets
GF	MuSyQ GF-series 16 m/10-day NDVI Dataset^25^	16 m	2018–2020/10-Day	https://www.scidb.cn/en/detail?dataSetId=841373295210135552
Sentinel	China 10 m Annual NDVI Maximum Dataset^30^	10 m	2016–2021/Yearly	https://www.resdc.cn/

AVHRR: Advanced Very High Resolution Radiometer

NOAA: National Oceanic and Atmospheric Administration

CDR: Climate Data Records

NDVI: Normalized Difference Vegetation Index

GIMMS: Global Inventory Monitoring and Modeling System

MODIS: Moderate-resolution Imaging Spectroradiometer

VIIRS: Visible Infrared Imaging Radiometer Suite

GF: Gao Fen
  Landsat data have great potential for exploitation as they provide a wealth of free time-series public historical imagery with a spatial resolution of 30 m^26–28^. Consequently, several NDVI data products have been developed based on Landsat^29^. For example, the “China annual maximum NDVI dataset (30 m,1986–2021)” relies on Landsat top of atmosphere (TOA) data as its source, computing NDVI throughout the year and selecting the maximum value to mosaic^30^. The “China annual maximum NDVI dataset (30 m, 2000–2020)” presented by Dong^31^ was generated by extracting NDVI values from valid Landsat surface reflectance (SR) observations throughout the year. Both linear interpolation and the Savitzky-Golay (SG) smoothing filter methods were used in this process^32^. However, there are some minor defects with these products in China’s coal bases. First, when using TOA data to calculate NDVI, the results are impacted by the atmosphere^33^. Secondly, the cloud cover would cause images missing during the most vigorous period of vegetation growth^34–36^. It is not appropriate for existing products to directly use data from other times to fill in the gaps. Furthermore, the problem of inconsistent time-phases for different images of Landsat are often ignored. This may cause notable mosaic lines in the NDVI data, as well as the annual maximum NDVI that falls considerably below the real value.
2. User-computed NDVI: Traditionally, users can compute NDVI through band math utilizing local software. Nevertheless, this approach is often time-consuming, especially when handling a large number of NDVI images. In contrast, Google Earth Engine (GEE) has become a leading platform for producing extensive time series of NDVI data^37–40^. For instance, Chen *et al*.^41^ presented the “Gap Filling and Savitzky-Golay filtering (GF-SG)” approach that effectively reconstructs 8-day NDVI time-series data using MODIS and Landsat data within GEE. Furthermore, GEE provides the qualityMosaic (QM) method for extracting the maximum value from image collections. However, it addresses missing pixels by incorporating data from other times.

In summary, due to satellite revisiting capability and cloud cover, there is missing data during the most vigorous vegetation growth period. The fusion of NDVI images with inconsistent imaging time causes some problems as follows. (1) The extracted annual maximum values of NDVI are significantly lower than the real values. (2) Visually, the NDVI image displayed a noticeable mosaic line.

In this paper, we proposed a GEE-based algorithm for automatically calculating the temporally-consistent annual maximum NDVI of China’s coal bases (Auto-NDVI_cb_). Auto-NDVI_cb_ is based on Landsat SR data and achieves online fast generation of seamless annual maximum NDVI data with long-time series, spatial continuity, and temporal consistency. High-quality vegetation monitoring data of coal bases provides solid data support for ecological environment monitoring in mining areas. The main contributions of this paper can be summarized below:

1. Auto-NDVI_cb_ solved the problem of the mosaic line caused by the lack of images during the vigorous vegetation growth and inconsistency in the acquiring time of nearby images. And is capable of producing temporally-consistent annual maximum NDVI of China’s coal bases.
2. The annual maximum NDVI dataset of 14 coal bases in China from 2013 to 2022^42^ is publicly available, which is closer to the reality of surface vegetation rather than just visual spatial smoothness. Compared with the existing annual maximum NDVI products and the current method, it shows better results at the scale of coal bases.
3. Auto-NDVI_cb_ can be fully automated in GEE, so the public dataset can be fast and automatically updated online in a continuous way based on the proposed Auto-NDVI_cb_ algorithm.

## Methods

### Study area

**Fig. 1:**
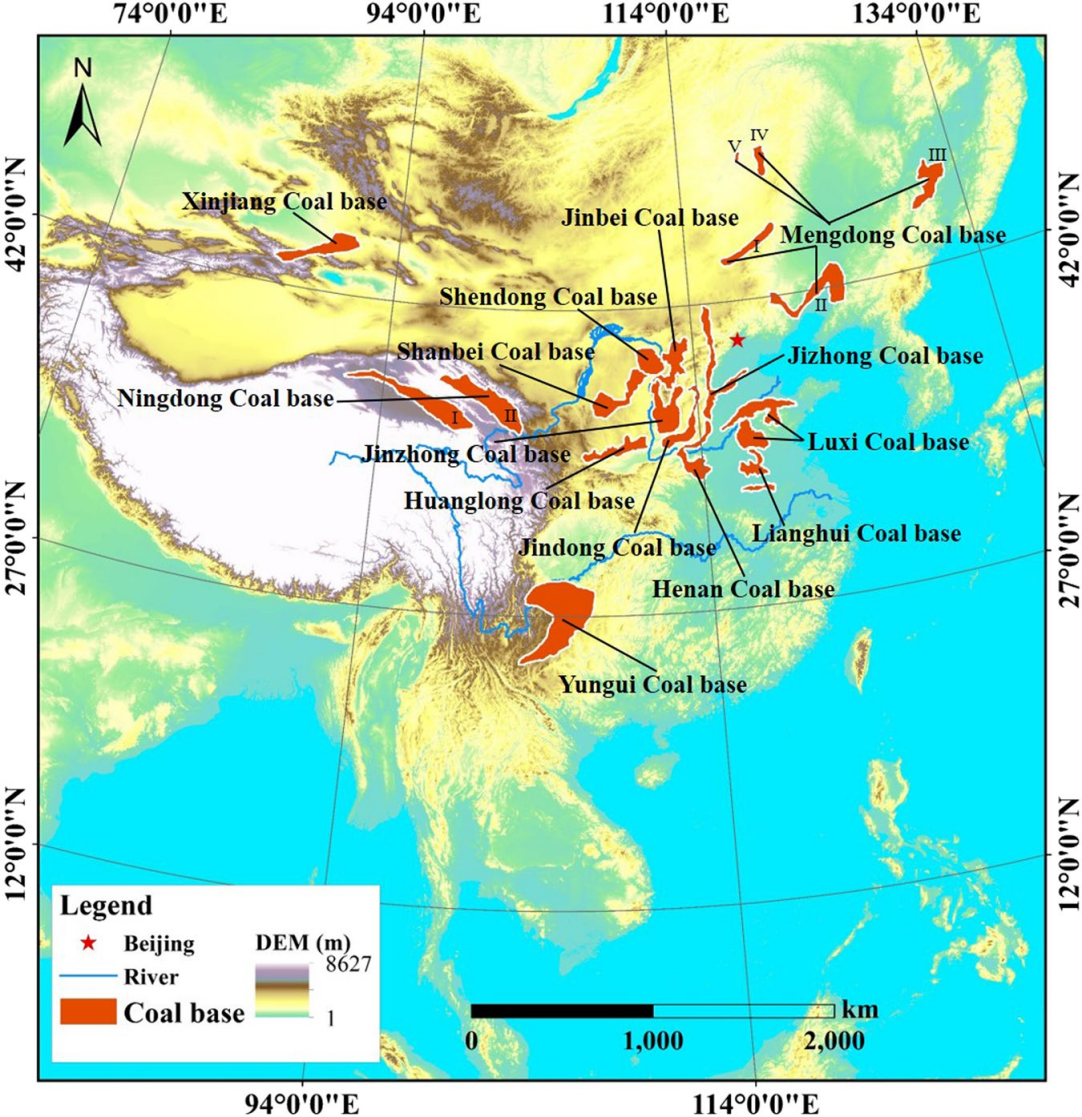
The location of 14 coal bases in China.

The NDVI data generated in this paper covers all of the 14 coal bases in China, i.e., Shendong (SD), Jinbei (JB), Jinzhong (JZ), Jindong (JD), Mengdong (MD), Yungui (YG), Henan (HN), Luxi (LX), Lianghuai (LH), Huanglong (HL), Jizhong (JIZ), Ningdong (ND), Shanbei (SB), and Xinjiang (XJ) coal bases (Fig. 1). These coal bases contain China’s major coal mines. At these coal bases, large-scale and high-intensity coal mining activities have resulted in significant vegetation changes in mining areas.

### Remotely sensed images

The remotely sensed images utilized in this study were the “USGS Landsat 8 Level 2, Collection 2, Tier 1”^43^. The remotely sensed images utilized in this study were the “USGS Landsat 8 Level 2, Collection 2, Tier 1”^43^. This dataset is atmospherically corrected surface reflectance data produced by the Landsat 8 OLI sensors using the Land Surface Reflectance Code (LaSRC) by the USGS Earth Resources Observation and Science (EROS) Center. The accuracy, precision, and uncertainty of the OLI surface reflectance data published by the USGS has been described quantitatively in detail in the reference^44^. It also has been demonstrated that the quality of OLI surface reflectance data generated by LaSRC allow scientists to provide more useful data products including the NDVI data^44^. It is publicly released on GEE with a spatial resolution of 30 m and a temporal resolution of 16 days (https://developers.google.com/earth-engine/datasets/catalog/landsat). The Worldwide Reference System (WRS) is used to identify a nominal Landsat scene by specifying the Path and Row numbers. The Path represents the descending orbit of the satellite. Row represents the location of a scene on the Path. In this paper, we use the notation ppprrr (i.e., Path = ppp and Row = rrr) according to the WRS system to designate each specific scene. For example, the scene with the number 126032 is identified by Path = 126 and Row = 032. Each scene approximately covers 170 km x 183 km area in China.

### The overview of the Auto-NDVI_cb_ algorithm

**Fig. 2:**
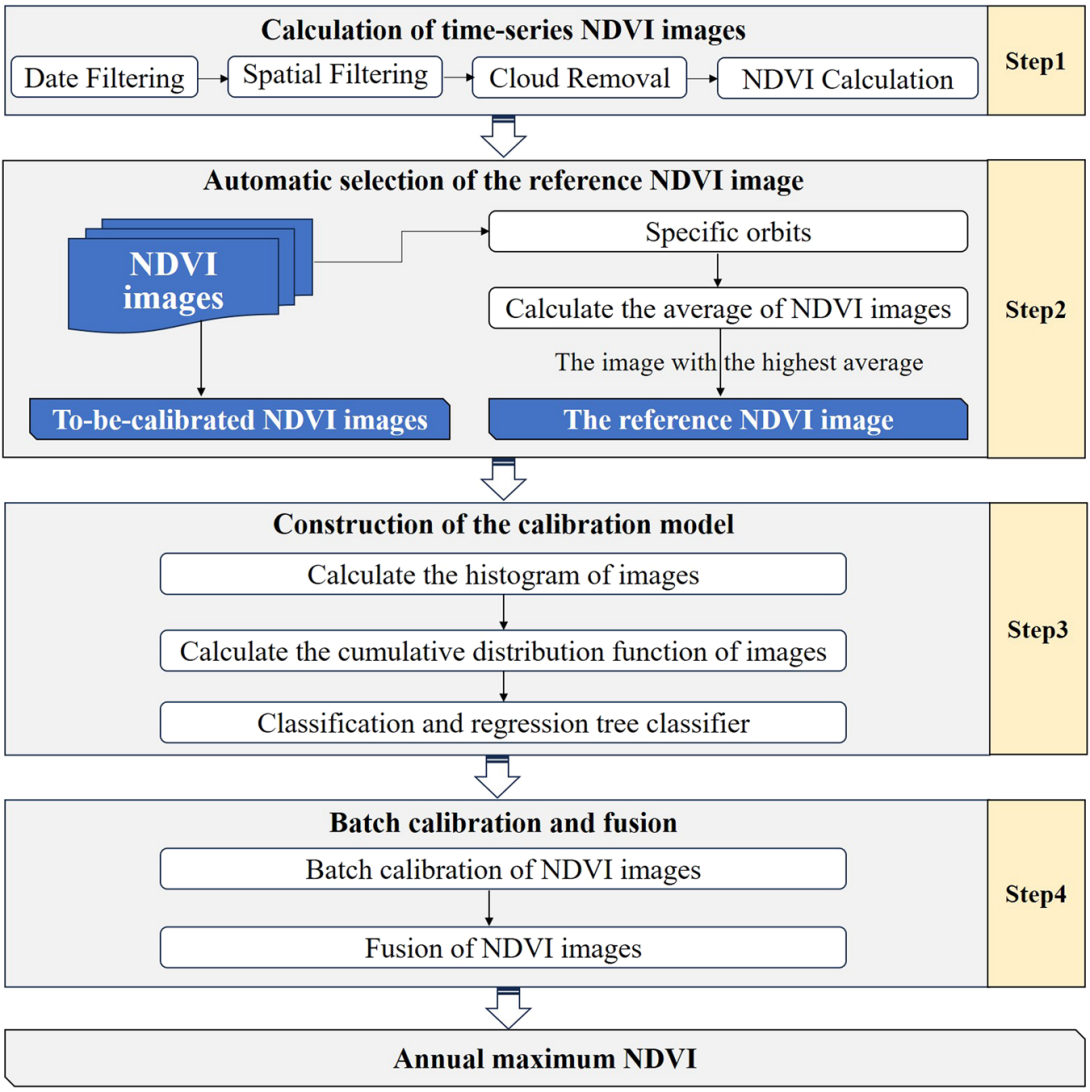
Procedure of the Auto-NDVI_cb_ algorithm.

Figure 2 illustrates the procedure of the proposed Auto-NDVI_cb_ algorithm, which can be conducted totally in GEE. First, images effectively observed throughout the whole year are filtered, and NDVI calculations are performed on these images. Second, an automated selecting function has been developed to choose the reference NDVI image for each coal base, which most closely characterizes the vegetation growth at its peak. Third, a calibration model is constructed, ensuring consistency in the NDVI cumulative distribution function across similar land cover. Finally, all the collected NDVI images covering the coal base are batch-calibrated based on the constructed model and fused to obtain the annual maximum NDVI data.

### Calculation of time-series NDVI

The time-series NDVI images were computed in GEE. Here is the description of the steps involved. First, the Landsat 8 SR dataset was loaded into GEE. Images falling within a designated period and specific geographical region were selected for further processing. Then, date filtering was applied to select images during the most vigorous vegetation growth period in the study area. For most coal bases, the date filtering parameter was set from April to October of each year to avoid extreme phenology. Given the limitations of cloud cover and Landsat revisit cycles, focusing only on summer season images would result in vacant pixel values in the NDVI results. An exception was made for the YG coal base. Frequent cloud coverage at the YG coal base throughout the year results in less valid data, so the date filtering parameter was set from January to December, thus ensuring the completeness of data coverage. The boundary of each coal base was used as a parameter for spatial filtering. Third, the cloud and its shadow were removed for each image after filtering. The cloud removal process for Landsat SR images in GEE focuses on clouds and cloud shadows^45^. The Quality Assessment (QA) band, which stores quality information for each image, was utilized. Pixels with quality information indicating “Cloud” and “Cloud Shadow” were filtered out using bitwise operations, creating a cloud mask. The “updateMask” function in GEE was then used to remove the cloud mask area and obtain cloud-free images^46^. There may be unscreened clouds (e.g., cirrus clouds) after the filtering. However, in our study, we did not find the impact of cirrus clouds on the results. Even if cirrus cloud pixels exist, the Auto-NDVI_cb_ algorithm will exclude them during the calculation of the maximum NDVI value. Finally, NDVI was computed for each image using the Eq. (1).

1 $${\rm{NDVI}}=\frac{{\rm{NIR}}-{\rm{RED}}}{{\rm{NIR}}+{\rm{RED}}}$$

where NIR represents the surface reflectance in the near-infrared band and RED represents the surface reflectance in the red band.

### Automatic selection of the reference NDVI image

**Fig. 3:**
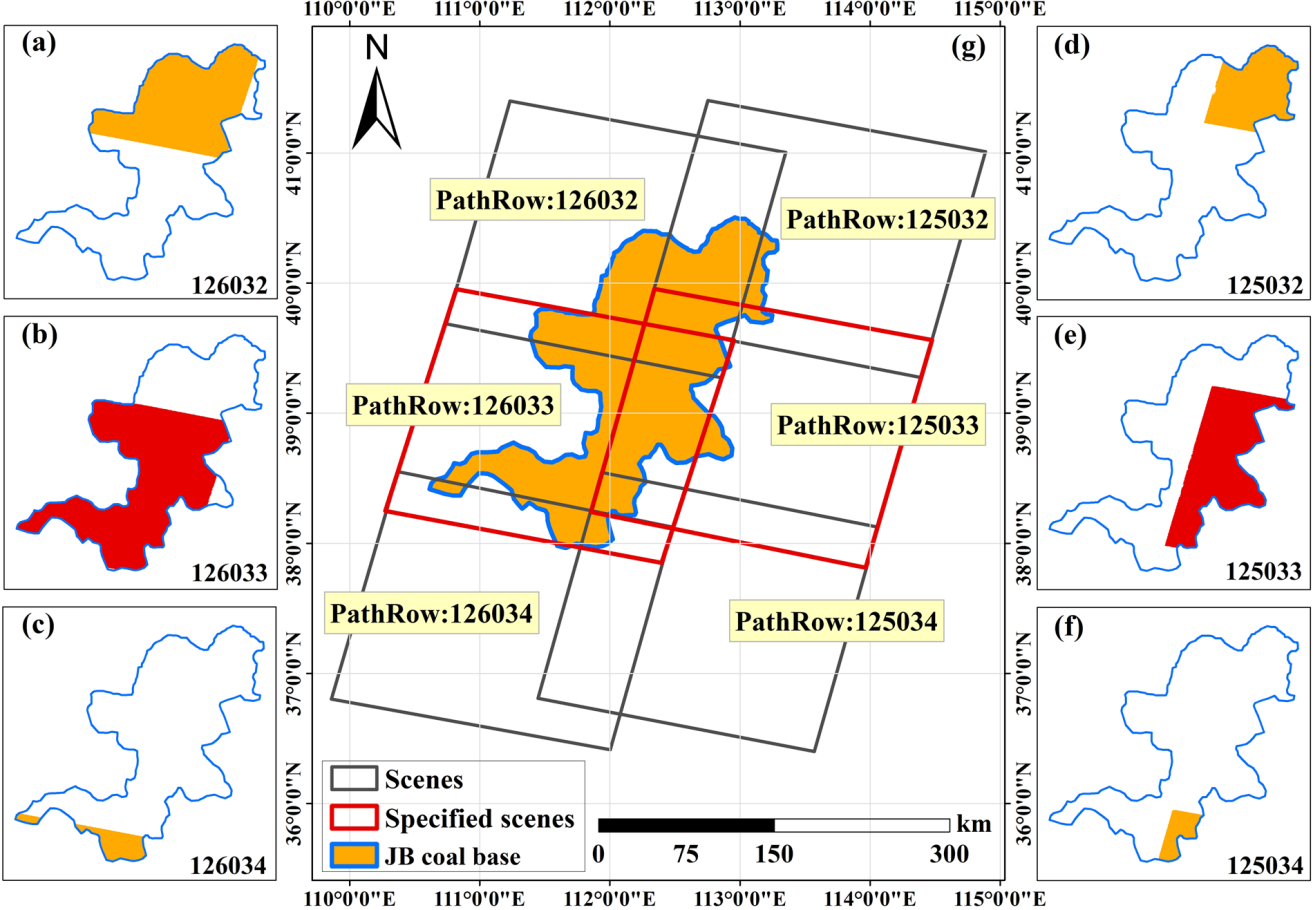
Coverage of Landsat scenes at JB coal base as an example.

The reference NDVI image for each coal base needs to most closely characterize the most vigorous vegetation growth in a year. Hence, the reference NDVI image is defined as the NDVI image with the highest average value among all the NDVI images of the coal base in a year. We designed an algorithm to automatically select the reference NDVI image from the NDVI collection in GEE. First, each coal base is covered by several scenes, and each scene covers an area of varying size. To ensure the sufficient coverage area of the reference NDVI image, 2-3 scenes were designated as selectable scenes at each coal base. For example, the JB coal base is covered by scenes of numbers 126032, 125032, 126033, 125033, 126034, and 125034 (Fig. 3). According to the coverage size, scene 126033 (Fig. 3(b)) and scene 125033 (Fig. 3(e)) were designated as selectable scenes at the JB coal base. Second, the images in the selectable scenes with over 40% clouds were removed to avoid severe missing pixels in the reference NDVI image after cloud removal. Finally, we calculate the average value of each NDVI image in the selectable scenes. The NDVI image with the highest average value is selected as the reference NDVI image.

### Construction of the calibration model

**Fig. 4:**
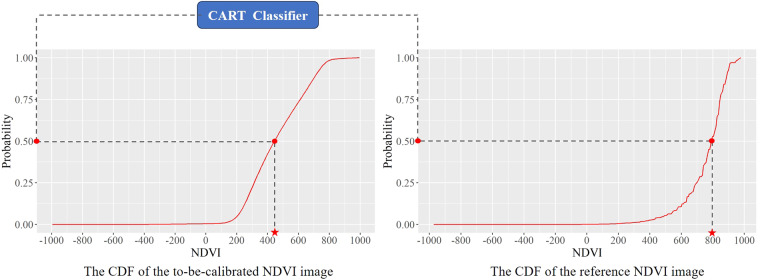
The calibration model (The NDVI values have been scaled up by a factor of 1,000).

Tobler’s First Law of Geography: All things are related, but nearby things are more related than distant things^42^. Simultaneously acquired Images of nearby areas generally have similar color tones, with very few abrupt changes in overall cliffiness. In other words, the cumulative probability distributions of contemporary NDVI images in nearby areas are similar. In light of this, a calibration model was constructed to harmonize the different time-phase NDVI images (called to-be-calibrated NDVI images) at all the scenes covering a coal base to align with the cumulative probability distribution of the reference NDVI image. Each coal base requires at least 4–9 scenes of images to be fully covered. There are 90 scenes at the 14 coal bases. Each scene has about 20 images per year. Therefore, at a minimum, about 1800 images in a year need to be processed. The construction of the calibration model includes the following steps:

1. The first step is to create a function that calculates the cumulative probabilities of both the reference NDVI image and the to-be-calibrated NDVI images. This function begins by computing the histogram of the NDVI image using the “ee.Reducer.histogram()” reducer. It then derives the Cumulative Distribution Function (CDF) of the NDVI image. Finally, the function outputs a feature set containing cumulative probability values corresponding to each NDVI image.
2. In the second step, a fitting model is constructed for the feature sets, mapping NDVI values from the to-be-calibrated NDVI images to the reference NDVI image. In this paper, the CART classifier function is used to build the fitting model on GEE. The parameter of the CART classifier function is set to “regression” to allow outputting continuous values from a standard regression. Any NDVI value “*a*_*i*_” in the to-be-calibrated NDVI image can find a corresponding NDVI value “*b*_*j*_” on the reference NDVI image so that the cumulative probabilities corresponding to “*a*_*i*_” and “*b*_*j*_” are equivalent (Fig. 4). The input data for this function includes the to-be-calibrated and the reference NDVI images.

### Batch calibration and fusion

The to-be-calibrated NDVI images at all the scenes covering a coal base were looped into the algorithm of constructing calibration models to get a calibrated NDVI image collection. Each calibrated NDVI was or was close to the annual maximum NDVI. The values of the calibrated NDVI image collection at each pixel were retrieved and sorted, and the median value was selected to get the annual maximum NDVI image. The function of median fusion can effectively avoid anomalous pixels. Considering the characteristics of discontinuous spatial distribution and the large geographic extent of each coal base, the algorithm was conducted for all 14 coal bases, respectively.

## Data Records

The annual maximum NDVI dataset^47^ of all 14 coal bases in China from 2013 to 2022 is available to the public at 10.6084/m9.figshare.c.6933322. It includes the following 14 compressed packages of 140 GeoTIFF images:

1. The annual maximum NDVI dataset of Henan (HN) coal base in China (2013–2022).
2. The annual maximum NDVI dataset of Jinbei (JB) coal base in China (2013–2022).
3. The annual maximum NDVI dataset of Xinjiang (XJ) coal base in China (2013–2022).
4. The annual maximum NDVI dataset of Jindong (JD) coal base in China (2013–2022).
5. The annual maximum NDVI dataset of Jinzhong (JZ) coal base in China (2013–2022).
6. The annual maximum NDVI dataset of Lianghuai (LH) coal base in China (2013–2022).
7. The annual maximum NDVI dataset of Luxi (LX) coal base in China (2013–2022).
8. The annual maximum NDVI dataset of Mengdong (MD) coal base in China (2013–2022).
9. The annual maximum NDVI dataset of Yungui (YG) coal bases in China (2013–2022).
10. The annual maximum NDVI dataset of Jizhong (JIZ) coal base in China (2013–2022).
11. The annual maximum NDVI dataset of Ningdong (ND) coal base in China (2013–2022).
12. The annual maximum NDVI dataset of Shanbei (SB) coal base in China (2013–2022).
13. The annual maximum NDVI dataset of Shendong (SD) coal base in China (2013–2022).
14. The annual maximum NDVI dataset of Huanglong (HL) coal base in China (2013–2022).

Each package represents the NDVI images in 10 years for each coal base, with a spatial resolution of 30 m. The geographic reference for all images is ESPG:4326 (WGS_1984). The values of the image range from −1 to 1.

## Technical Validation

### Accuracy of reconstructing the annual maximum NDVI images

Experiments were conducted to assess the accuracy of the Auto-NDVI_cb_ algorithm by assuming the absence of the annual maximum NDVI image. Since a single sensor was used as a data source, the maximum NDVI true value acquired by that sensor should be used as validation data. The nearby image that has the same imaging time as the reference NDVI image also closely represents the annual maximum NDVI. This image is regarded as the “True image”, and assumed missing. Auto-NDVI_cb_ algorithm is conducted to reconstruct the annual maximum NDVI (“Predicted image”). The images used for accuracy validation are independent and high-quality data. The Root Mean Square Error (RMSE) between the “Predicted image” and the “True image” is calculated to verify the accuracy as Eq. (2). Here we use the example of the JB coal base in 2020 to provide a detailed illustration of the experiments. As shown in Fig. 3, the image acquired on 24^th^ August with PathRow = 126033 has been selected as the reference NDVI image, most closely characterizing the most vigorous vegetation growth in 2020. The image with PathRow = 126032 was also acquired on 24^th^ August, and also closely represented the annual maximum NDVI. Hence, the image (PathRow = 126032, 24^th^ August) was regarded as the “True image” and removed to simulate the absence of the annual maximum NDVI image. The Auto-NDVI_cb_ algorithm was conducted to predict the annual maximum NDVI image based on the reference NDVI image (PathRow = 126033, 24^th^ August). Finally, the RMSE was calculated. In this study, the same experiments were carried out for all 14 coal bases to validate the accuracy.

2 $${\rm{RMSE}}=\frac{1}{n}\sqrt{{\mathop{\sum }\limits_{i=1}^{n}[{{\rm{value}}}_{{Predicted}}({{\rm{x}}}_{{\rm{i}},}{{\rm{y}}}_{{\rm{i}}})-{{\rm{value}}}_{{True}}({{\rm{x}}}_{{\rm{i}},}{{\rm{y}}}_{{\rm{i}}})]}^{2}}$$

where n represents the number of pixels in the image; ${{\rm{value}}}_{Predicted}({{\rm{x}}}_{{\rm{i}},}{{\rm{y}}}_{{\rm{i}}})$ represents the NDVI value of the pixel at position x, y in the “*Predicted* image”; ${{\rm{value}}}_{True}({{\rm{x}}}_{{\rm{i}},}{{\rm{y}}}_{{\rm{i}}})$ represents the NDVI value of the pixel at position x, y in the “*True* image”.

**Fig. 5:**
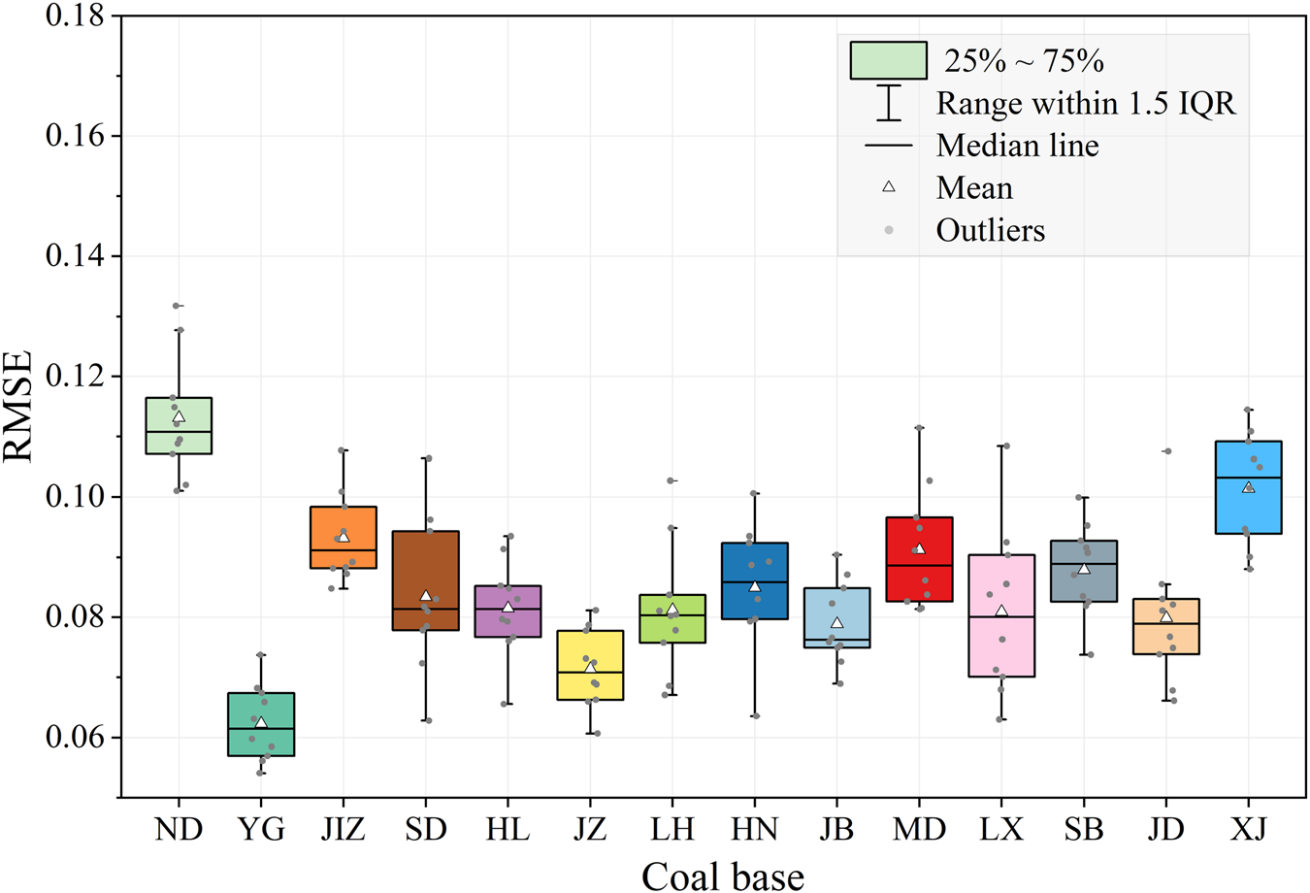
Distribution of RMSE in 14 coal bases from 2013 to 2022.

Table 2 shows the RMSE of the Auto-NDVI_cb_ algorithm year by year in each coal base. The bold values indicate the maximum and minimum values of RMSE from 2013–2022 for each coal base. The algorithm produced an average RMSE of 0.087 across the 14 coal bases from 2013 to 2022. The algorithm had varying accuracy in different coal bases. It suggests that the highest accuracy is present in the YG coal base with an average RMSE of 0.062, followed by the JZ and JB coal base at 0.071 and 0.079, respectively. The average RMSEs for the JD, LH, LX, HL, SD, HN, and SB coal bases all ranged between 0.080 and 0.088. By contrast, the ND coal base displayed the lowest accuracy, with the highest average RMSE of 0.113. In terms of accuracy in specific years, the YG coal base achieved the lowest RMSE of 0.054 in 2022, while the ND coal base had the highest RMSE of 0.132 in 2015. The YG coal base belongs to the broad-leaved evergreen forest area, where the land cover is dominated by vegetation. Moreover, the vegetation grew vigorously, and the land cover between the scenes was close to each other. On the contrary, the ND coal base belongs to the plateau-continental climate area, with sparse surface vegetation and large heterogeneity of land covers between different scenes, but the RMSE of 0.132 in the ND coal base is also acceptable. The maximum inter-annual differences of RMSE vary from 0.020 to 0.045 with different coal bases, with an average of 0.031. In terms of all the coal bases, the average RMSE varies from 0.081 to 0.093 with different years. Figure 5 displays the distribution of RMSE for 14 coal bases from 2013 to 2022. Outliers were only observed in the LH and JD coal bases, exhibiting RMSE values of 0.103 and 0.108 correspondingly. Taken together, Fig. 5 and Table 2 show that the accuracy of the Auto-NDVI_cb_ algorithm fluctuates little from year to year and remains stable. Overall, the Auto-NDVI_cb_ algorithm exhibits a satisfactory performance across the 14 coal bases.

**Table 2:** RMSE of the Auto-NDVI_cb_ algorithm in 14 coal bases from 2013 to 2022.

	YG	JZ	JB	JD	LH	LX	HL	SD	HN	SB	MD	JIZ	XJ	ND	Average
2013	**0.074**	0.066	0.085	0.077	0.076	0.070	0.091	0.072	0.092	**0.100**	0.103	0.088	0.111	0.110	0.087
2014	0.066	0.078	0.087	**0.108**	**0.103**	**0.108**	0.085	**0.063**	0.080	0.092	**0.111**	0.101	**0.088**	0.128	0.093
2015	0.067	0.069	0.075	0.082	0.080	0.092	**0.066**	0.096	**0.064**	0.095	0.083	**0.108**	**0.114**	**0.132**	0.087
2016	0.057	0.066	0.076	**0.066**	0.069	0.076	0.083	**0.106**	0.083	0.084	0.086	0.094	0.106	0.112	0.083
2017	0.068	0.069	0.073	0.075	0.081	0.071	**0.094**	0.082	0.094	0.087	0.095	0.098	0.105	0.115	0.086
2018	0.060	0.079	0.077	0.083	**0.067**	0.068	0.079	0.078	0.079	0.083	0.097	0.087	0.109	0.109	0.082
2019	0.058	**0.061**	0.082	0.081	0.095	0.090	0.076	0.083	**0.101**	**0.074**	0.082	**0.085**	0.101	0.107	0.084
2020	0.056	0.073	0.075	0.068	0.078	**0.063**	0.085	0.079	0.089	0.091	0.091	0.093	0.095	**0.101**	0.081
2021	0.063	**0.081**	**0.090**	0.074	0.080	0.084	0.077	0.094	0.089	0.082	**0.081**	0.089	0.094	0.116	0.085
2022	**0.054**	0.073	**0.069**	0.085	0.084	0.086	0.080	0.081	0.080	0.093	0.084	0.088	0.090	0.102	0.082
Average	0.062	0.071	0.079	0.080	0.081	0.081	0.082	0.083	0.085	0.088	0.091	0.093	0.101	0.113	0.087
Max-Min	0.020	0.020	0.021	0.041	0.036	0.045	0.028	0.044	0.037	0.026	0.030	0.023	0.026	0.031	0.031

### Applicability of the Auto-NDVI_cb_ algorithm to the 14 coal bases

The theoretical basement of Auto-NDVI_cb_ is Tobler’s First Law of Geography. So, when the to-be-calibrated NDVI image is far away from the reference NDVI image, the accuracy of Auto-NDVI_cb_ may be lower. Experiments were conducted to assess the applicability of the Auto-NDVI_cb_ algorithm at the scale of coal bases and measure the accuracies of different scenes. The JB coal base is also taken as an example to illustrate the experiments, and the accuracies of scenes 125032, 125033, 125034, and 126034 were measured. First, the image that is closest in each scene to the time of the reference NDVI image was selected as the “*True* image”. Second, the *True* image of each scene was removed to simulate the absence of the annual maximum NDVI image, respectively. Third, the reconstructed annual maximum NDVI images (called “*Predicted* image”) were produced by the Auto-NDVI_cb_ algorithm. Finally, the RMSE between the *True* images and the *Predicted* images were calculated, respectively. Table 3 shows the accuracy of each scene covering the 14 coal bases. We define a term called “image distance”. The image distance is the sum of the number of Path and Row differences between the to-be-calibrated NDVI image and the reference NDVI image as Eq. (3).

3 $${\rm{image}}\,{\rm{disatnace}}=|{{\rm{Path}}}_{cal}-{{\rm{Path}}}_{ref}|+|{{\rm{Row}}}_{cal}-{{\rm{Row}}}_{ref}|$$

where Path_*cal*_ represents the Path number of the to-be-calibrated NDVI image; Row_*cal*_ represents the Row number of the to-be-calibrated NDVI image; Path_*ref*_ represents the Path number of the reference NDVI image; Row_*ref*_ represents the Row number of the reference NDVI image.

**Table 3:** Accuracy of each scene of 14 coal bases.

Coal base	Reference NDVI Image	Image distance	Scene	RMSE	Coal base	Reference NDVI Image	Image distance	Scene	RMSE
JD	125034	1	125035	0.068	SD	127033	1	127032	0.079
		2	125036	0.082			1	126033	0.081
		2	126035	0.086			2	126032	0.088
LH	122037	1	122036	0.078	HN	12436	1	124035	0.089
		1	121037	0.091			1	125036	0.091
		2	121036	0.094			1	124037	0.105
SB	127033	1	127034	0.091	JZ	126034	1	126035	0.073
		2	128034	0.105			1	126033	0.075
		3	129034	0.112			1	125034	0.083
							2	125035	0.085
JB	126033	1	125033	0.075	JIZ	124034	1	124033	0.093
		1	126032	0.077			1	123034	0.094
		1	126034	0.079			1	124035	0.103
		2	125034	0.082			2	125033	0.106
		2	125032	0.084			2	123033	0.107
							3	125032	0.120
LX	122036	1	122035	0.063	ND	133034	1	134034	0.102
		1	123036	0.077			1	133033	0.106
		1	121036	0.078			1	132034	0.107
		2	122034	0.077			2	133035	0.113
		2	123035	0.083			2	134033	0.118
		2	121035	0.085			2	132035	0.114
		3	123034	0.095			2	135034	0.127
		3	121034	0.096			3	135033	0.133
XJ	141029	1	141030	0.095	YG	128041	1	128042	0.054
		1	142029	0.098			1	128040	0.071
		2	142030	0.106			1	129041	0.073
		2	143029	0.120			2	128043	0.073
		3	143030	0.122			2	129040	0.067
		3	144029	0.131			2	129042	0.075
		4	144030	0.140			3	129043	0.090
HL	127035	1	127036	0.085	MD	11428	1	114027	0.091
		1	126035	0.086			1	114029	0.090
		1	128035	0.088			1	115028	0.092
		2	128036	0.096			2	115027	0.098
		2	126036	0.097			2	115029	0.100
		2	129035	0.099			2	116028	0.103
		3	129036	0.112			3	116029	0.114

**Fig. 6:**
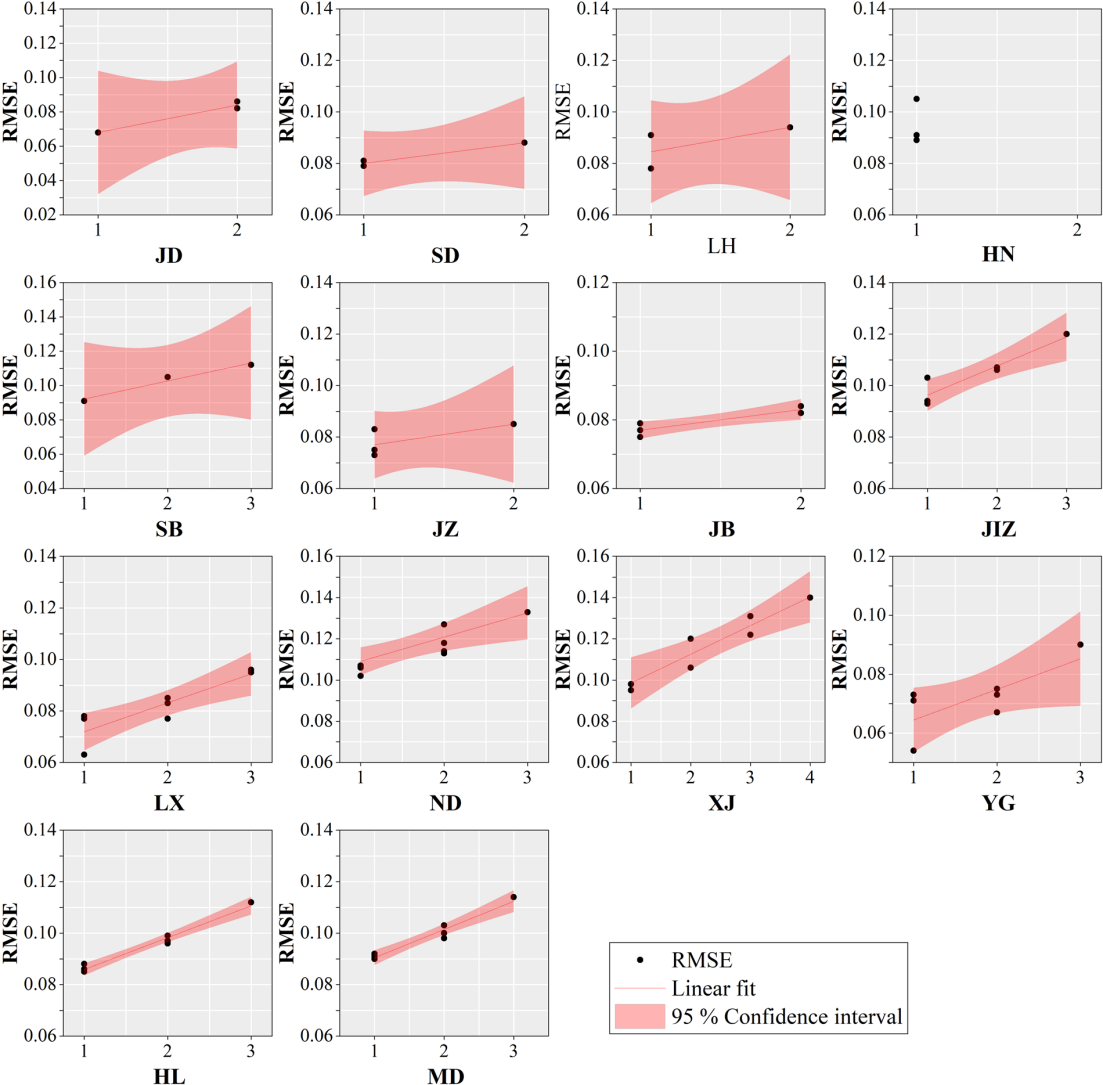
The variation of RMSE with image distance. (**a**-**n**) for 14 coal bases.

Figure 6 shows the variation of RMSE with image distance. Among all the scenes, the lowest RMSE is scene 122035 of LH coal base, with an image distance of 1 and an RMSE of 0.063. The highest RMSE is scene 144030 of the XJ coal base, with an image distance of 4 and an RMSE of 0.140. In terms of specific coal bases, for example, in JD coal base, the image distance of scene number 125035 is 1, and the RMSE is 0.068; the image distance of scene numbers 125036 and 126035 is 2, and the RMSE is 0.082 and 0.086, respectively. All 14 coal bases show that the farther the distance between the to-be-calibrated image and the reference NDVI image, the higher the RMSE. The farthest image distance is 4, and the accuracy of the algorithm is still acceptable (0.140).

### Impact of Imaging Time on Accuracy

**Fig. 7:**
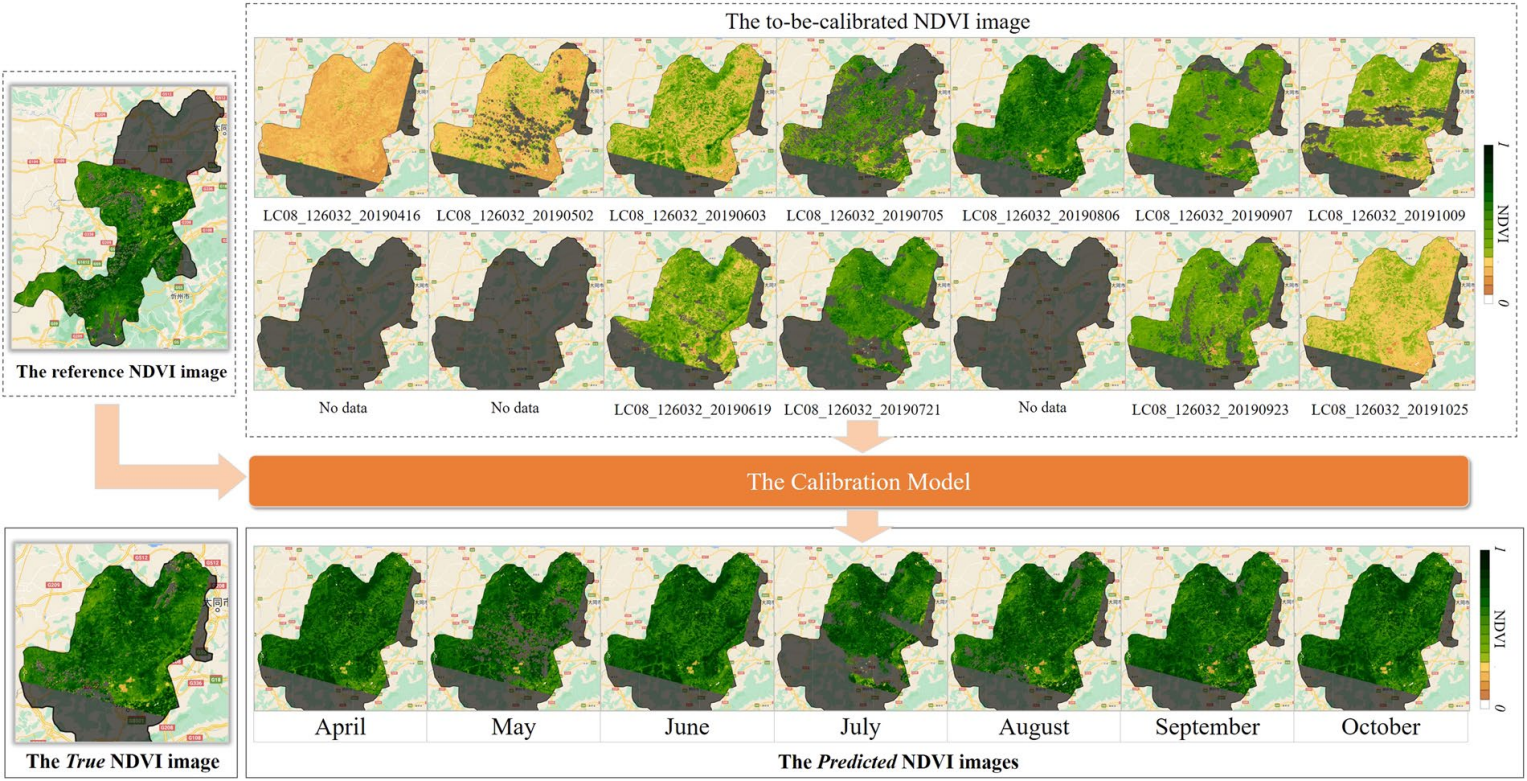
Calibration of NDVI images month by month (JB coal base as an example).

This experiment aims to assess the accuracy of the Auto-NDVI_cb_ algorithm for to-be-calibrated NDVI images from different months. The experimental process is illustrated in Fig. 7 (JB coal base as an example). The nearby image that has the same imaging time as the reference NDVI image is regarded as the “*True* image” and removed. The to-be-calibrated image in each month is calibrated by Auto-NDVI_cb_ to produce the “*Predicted* image” (e.g., the April and May in Fig. 7). If there is not only one image (usually two images) in a month, the images in this month are calibrated respectively, and is fused by “median” function in GEE to produce the “*Predicted* image” in this month (e.g., the June and July in Fig. 7). Finally, the RMSE between the *True* image and the *Predicted* image for each month is calculated. This experiment has been carried out for each coal base, respectively.

**Fig. 8:**
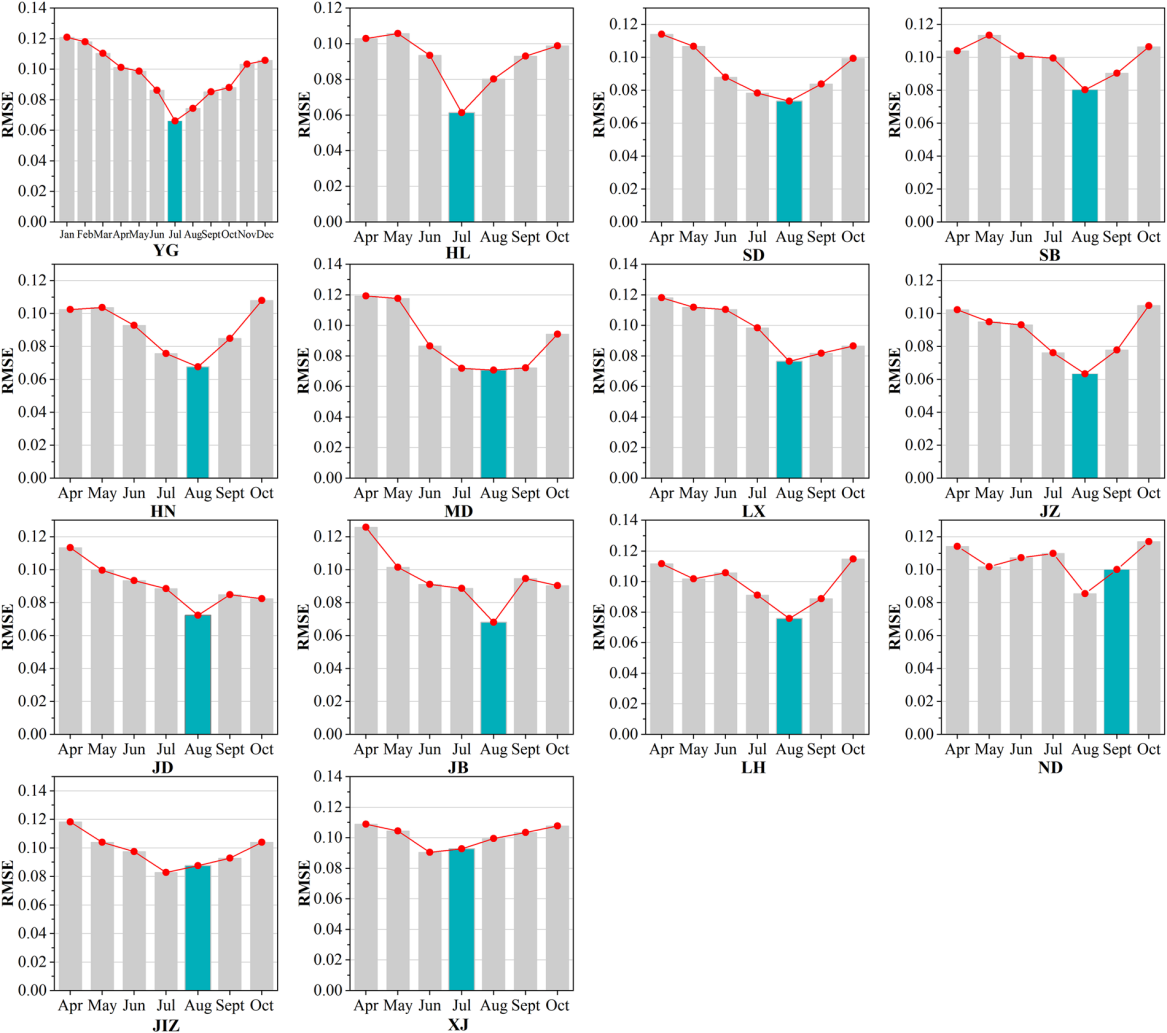
RMSE for different months in 14 coal bases. The cyan bar denotes the month of the reference NDVI image.

Figure 8 shows the variation of RMSE with months at each coal base. The RMSE showed an obvious trend of decreasing and then increasing. The months with the highest accuracy were mainly July and August. The RMSE of YG, JIZ, and HL coal bases was lowest in July, and the RMSE of SD, JZ, LH, HN, JB, MD, LX, SB, and ND coal bases was lowest in August. RMSE in XJ was lowest in June, and RMSE in JD was lowest in September.

The month with the lowest RMSE usually coincided with the month in which the maximum NDVI was recorded, i.e., the month of the reference NDVI image. For instance, in terms of the JZ coal base, the reference NDVI image was acquired on 24^th^ August and the lowest RMSE of 0.063 also appeared in August. Similarly, the reference NDVI image for the YG coal base was acquired on 11^th^ July, and the lowest RMSE was present in July with 0.061. This trend is also observed for the HL, SD, JZ, LH, HN, JB, MD, LX, SB, and JD coal bases. However, there are some exceptions. For example, the ND coal base does not display the lowest RMSE in September, the month of the reference NDVI image. This misalignment can be attributed to the date of reference NDVI image, i.e., 1^st^ September, which is close to August for the lowest RMSE. Although the month with the lowest RMSE in the XJ and JIZ coal bases did not exactly align with the month of the reference NDVI image, the difference between the lowest RMSE and the RMSE in the month of the reference NDVI image is small (XJ: 0.090 vs. 0.093; JIZ: 0.083 vs.0.088). These results suggest that the accuracy of the Auto-NDVI_cb_ is related to the difference in imaging time between the to-be-calibrated NDVI image and the reference NDVI image. However, even when the difference in imaging time is the largest, for example, the to-be-calibrated NDVI image in January (YG coal base) or April (JB coal base), the largest RMSE is 0.121 (YG coal base) and 0.126 (JB coal base). It indicates that the imaging time of the to-be-calibrated NDVI image has an effect on the accuracy, but the effect is limited and acceptable.

### Comparison with other NDVI datasets and methods

In this experiment, the annual maximum NDVI computed by the Auto-NDVI_cb_ algorithm was compared with NDVI images from the publicly available dataset and NDVI computed by the qualityMosaic method in GEE. The two publicly available datasets are the “32-Day NDVI Composite”^48^ produced by USGS (referred to as the “USGS” dataset) and the “China annual maximum NDVI dataset (30 m, 2000–2020)”^31^ (referred to as the “CNMD” dataset). The “USGS” dataset is made from Tier 1 orthorectified scenes, using the computed top-of-atmosphere (TOA) reflectance. These composites are created from all the scenes in each 32 days beginning from the first day of the year and continuing to the 352nd day of the year. The last composite of the year, beginning on day 353, will overlap the first composite of the following year by 20 days. All the images from every 32 days are included in the composite, with the most recent pixel as the composite value, which is publicly available on GEE. In this study, we extracted the annual maximum NDVI at the pixel scale. The “CNMD” dataset utilizes all Landsat5/7/8/9 remote sensing data throughout the year to obtain the maximum value of NDVI in one year for each pixel from 2000 to 2020 using series data preprocessing and data smoothing. The dataset is produced based on the GEE cloud computing platform and publicly released in the National Ecosystem Science Data Center. The dataset has a spatial resolution of 30 m and a temporal resolution of years.

**Fig. 9:**
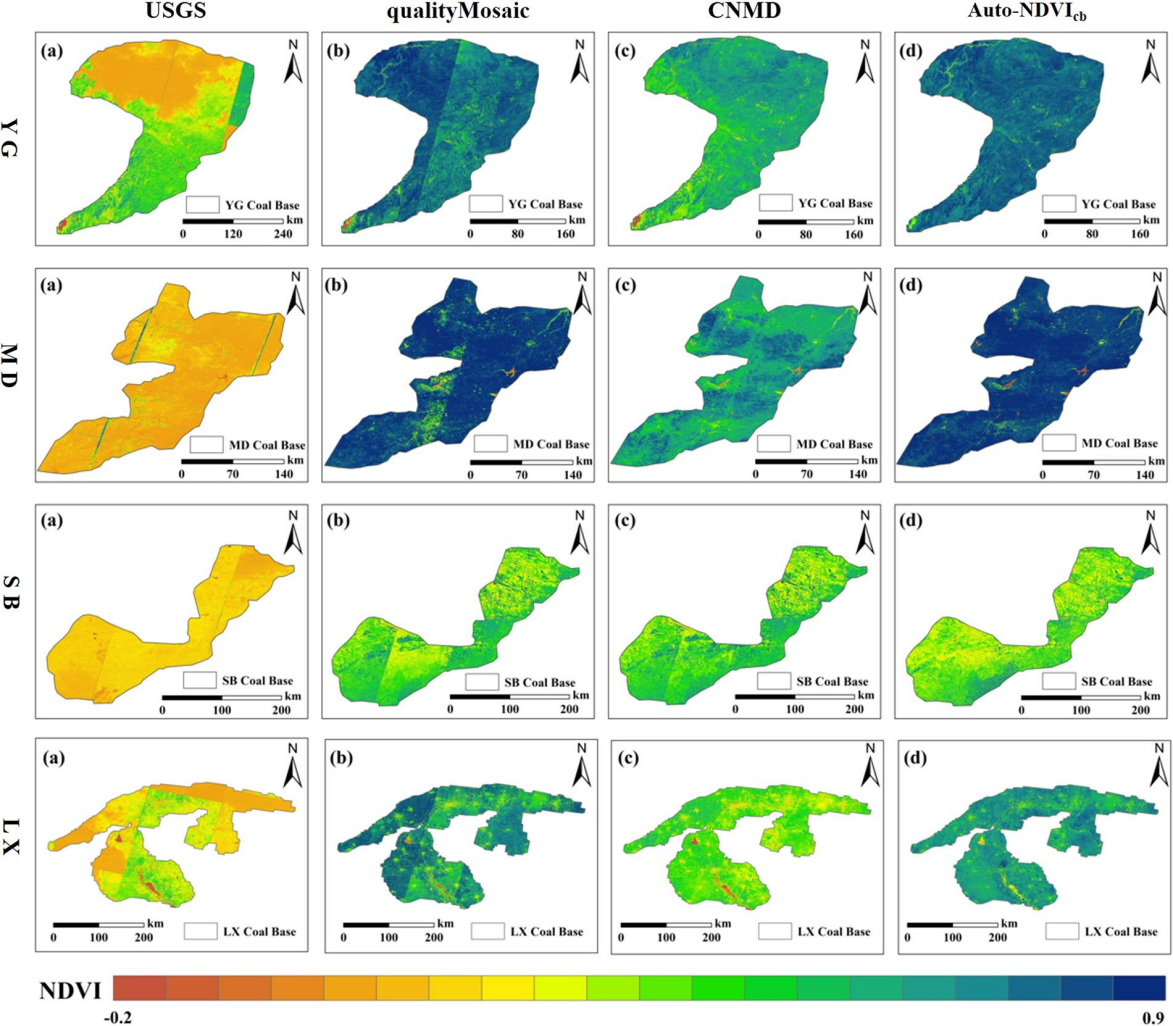
Annual maximum NDVI of the YG, MD, SB, LX coal bases. (**a**) The “USGS” dataset; (**b**)The qualityMosaic method; (**c**) The “CNMD” dataset; (**d**) The Auto-NDVI_cb_ algorithm.

Figure 9 shows the annual maximum NDVI results for the YG, MD, SB, and LX coal base from the “USGS” dataset, the qualityMosaic method, the “CNMD” dataset, and the Auto-NDVI_cb_ algorithm. In Fig. 9(a,b), the mosaic lines were noticeable. For the “USGS” dataset, the TOA data was used for calculating NDVI, which lacked atmospheric correction. Hence, the NDVI values in Fig. 9(a) were significantly lower than others. The qualityMosaic method can calculate NDVI using Landsat SR data, but it usually suffers from the problem of missing data during the most vigorous vegetation growth period due to the satellite revisiting capability and cloud cover. The missing data were replaced by data at other times in the qualityMosaic method. Consequently, the annual maximum NDVI images exhibited distinct mosaic lines, and some areas had considerably lower NDVI values compared to the real annual maximum NDVI values. Figure 9(c) displays the annual maximum NDVI results from the “CNMD” dataset. While this dataset shows slight traces of mosaic lines in the visualization, the overall NDVI values were lower. To address the problems of missing data during the peak of vegetation growth, time-series images were interpolated and subjected to SG filtering. However, this approach could not fully recover the annual maximum NDVI, leading to lower NDVI values overall. Figure 9(d) shows the annual maximum NDVI results generated by the Auto-NDVI_cb_ algorithm. These results do not show mosaic lines in the visualization, and the values are closer to the real annual maximum NDVI values. In comparison, the Auto-NDVI_cb_ algorithm is capable of producing annual maximum NDVI with better temporal consistency at the scale of coal bases.

**Fig. 10:**
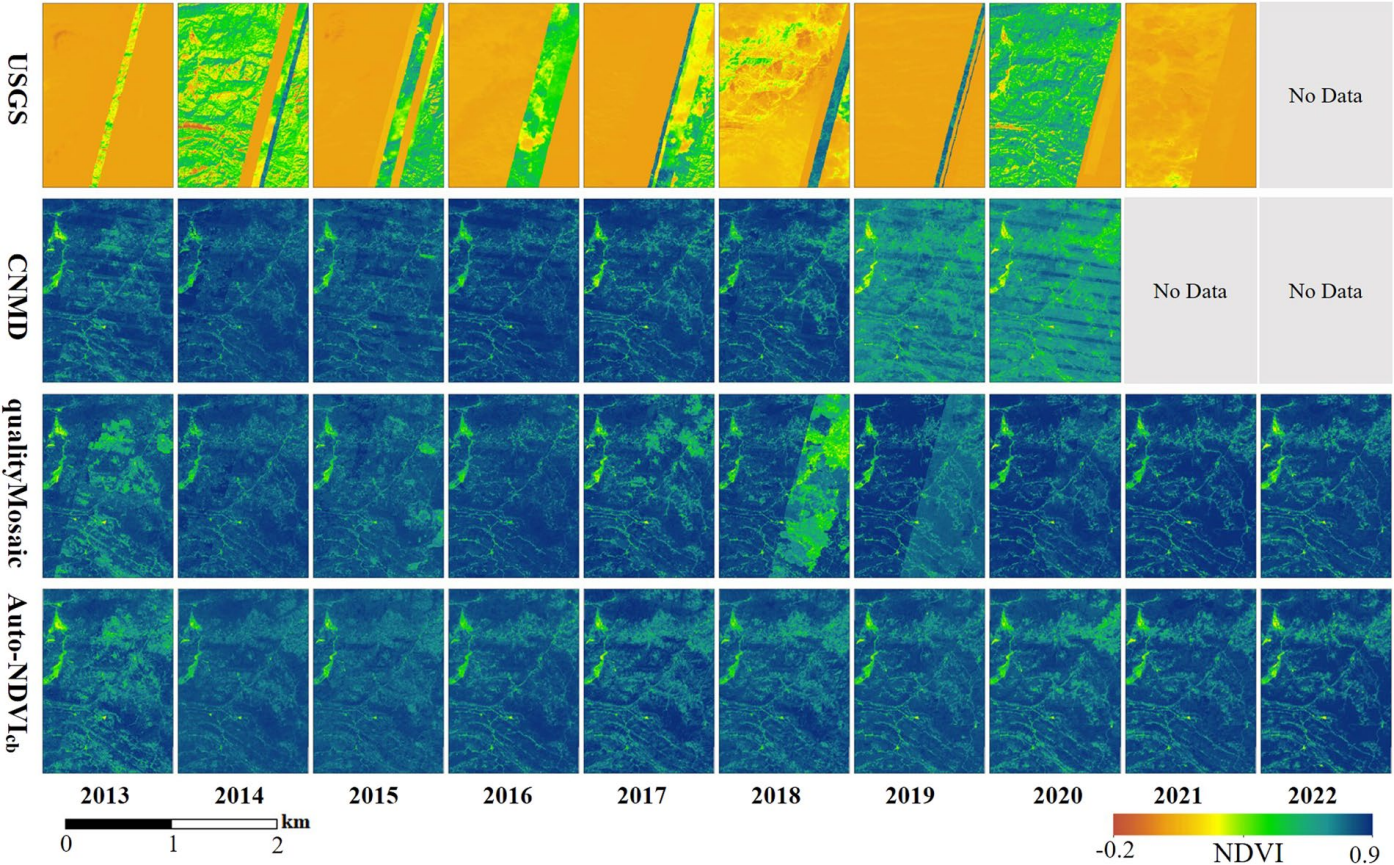
The annual maximum NDVI data in time series from different datasets and methods.

We conducted a detailed comparison of annual maximum NDVI data in time series from different datasets and methods using a sample area within the YG coal base, as shown in Fig. 10. The USGS dataset is available until 2021. However, its NDVI values were significantly lower than those of the other three datasets and exhibited noticeable mosaic lines. The qualityMosaic method resulted in an NDVI that highlights a distinct mosaic line for the years 2018 and 2019. The CNMD dataset is available until 2020. In terms of visualization, it displays horizontal stripes in NDVI images. Moreover, the NDVI values for 2019 and 2020 were abnormally lower in comparison to the first six years.

**Fig. 11:**
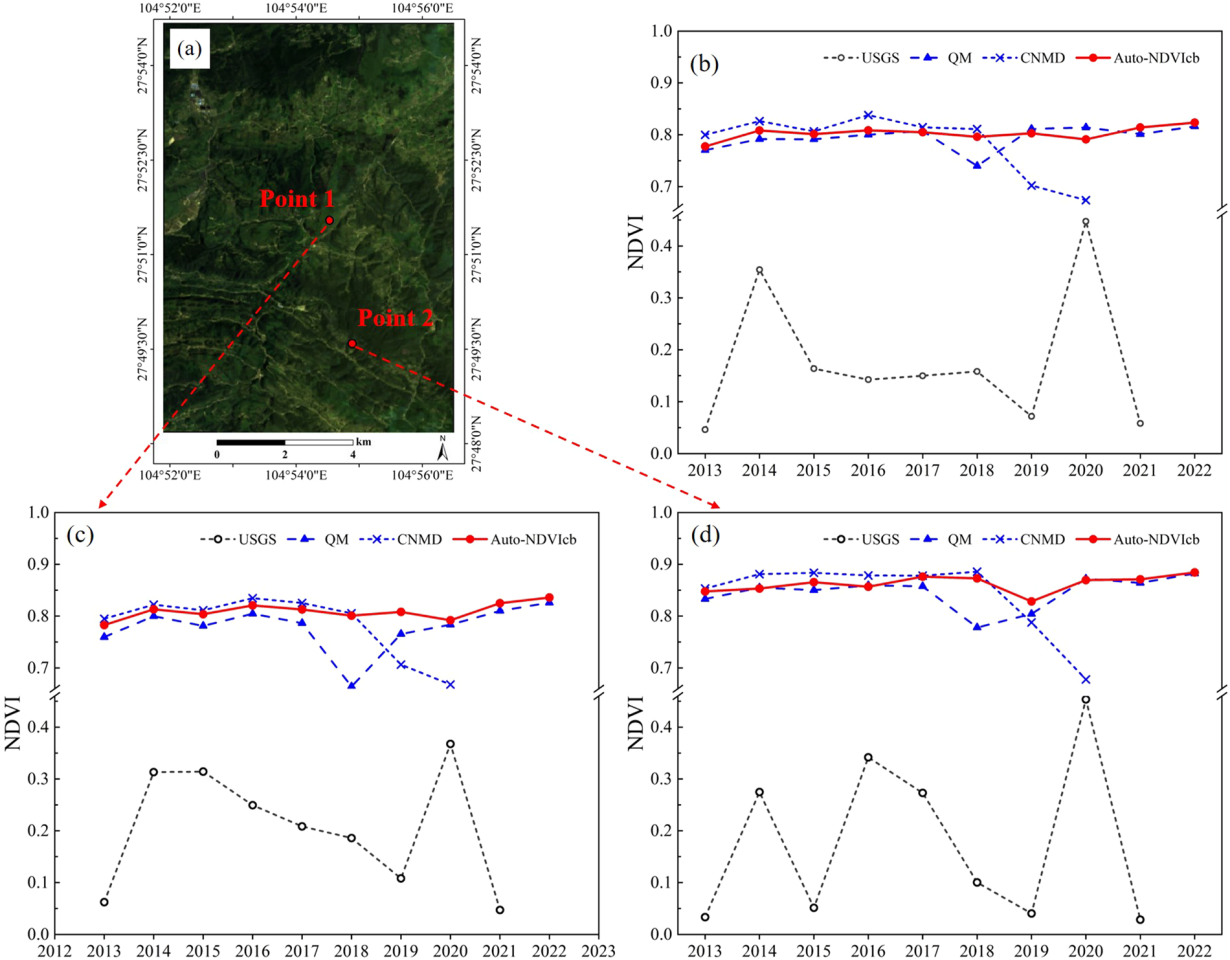
The time series plots of NDVI for different datasets. (**a**) The true-color composite of remote sensing images on the sample area; (**b**) Mean values of NDVI in the sample area; (**c**) Time-series NDVI at point 1; (**d**) Time-series NDVI at point 2.

To further compare different NDVI datasets, two sample points were randomly chosen and the NDVI time-series plots for the “USGS” dataset, qualityMosaic method, “CNMD” dataset, and Auto-NDVI_cb_ algorithm were plotted (Fig. 11). Combined with Fig. 10, it can be seen that Fig. 11(c) “QM” data in 2018 NDVI values plummeted may not attribute to vegetation damage, but the absence of remote sensing image at the moment of the most vigorous growth of vegetation. The results suggest that the Auto-NDVI_cb_ algorithm maintained consistently stable annual maximum NDVI values, even during years when abnormal NDVI values were produced by the “CNMD” dataset and qualityMosaic method. Furthermore, the changing pattern of NDVI generated by the Auto-NDVI_cb_ algorithm over years is consistent with that of the NDVI from the “CNMD” dataset and the qualityMosaic method. Consequently, these results demonstrate that the Auto-NDVI_cb_ algorithm reliably delivers the annual maximum NDVI and displays better consistency over distinct years.

### The superiority of the Auto-NDVI_cb_ algorithm

In this paper, a new algorithm for automatically producing the annual maximum NDVI of China coal bases for GEE (Auto-NDVI_cb_) was proposed. The problem of the mosaic line caused by the absence of images with maximum NDVI and inconsistency in the acquiring time of adjacent images was solved. The Auto-NDVI_cb_ shows the following superiorities.

1. Auto-NDVI_cb_ is only based on Landsat as the data source and does not require data from other satellite sensors (e.g., MODIS) to fuse and interpolate the missing NDVI data with different spatial resolutions. Sensors such as MODIS or AHI provide data that are suitable for spatially homogeneous vegetation over a range of several hundred meters. However, few scenarios meet this vegetation requirement, especially in mining areas. In this paper, we would like to provide an annual maximum NDVI with higher resolution and data quality to facilitate a more accurate analysis of the ecological changes in the mining area. Sensor fusion may bring some new problems. The NDVI calculated by different sensors is inconsistent due to spatial^49^, temporal^50^, radiometric, and spectral factors. The single data source reduces the systematic bias caused by heterogeneous sensors as well as the loss of data detail information caused by low resolution^50,51^. The Auto-NDVI_cb_ algorithm proposed in this paper avoids these problems by using a single sensor and finishing the task of generating annual maximum NDVI with satisfying accuracy.
2. Auto-NDVI_cb_ can be automatically and completely performed in GEE, including the image filtering, cloud removal, NDVI calculation, selection of the reference NDVI image, construction of the calibration model, batch calibration, and the last fusion to generate annual maximum NDVI. While the calibration of a single image can be achieved using software like The Environment for Visualizing Images (ENVI) or ERDAS IMAGINE, it becomes cumbersome when dealing with study areas covered by multiple satellite scenes and multiple time-phase images. For instance, the YG coal base required data from at least 7 scenes, totalling around 168 images per year for a single coal base. When the research was extended to 14 coal bases and carried out for more than ten years, it was impractical to download the images, then filter the reference NDVI images from many images, and calibrate a large number of NDVI images.

### Future of the Auto-NDVI_cb_ algorithm

While the Auto-NDVI_cb_ algorithm has achieved satisfactory accuracy in producing the annual maximum NDVI of the 14 coal bases in China, it still has some limitations to address in the future.

First, the algorithm was based on Tobler’s First Law of Geography and the consistency of the cumulative distribution probability function of similar features. However, it’s important to recognize that features from different scenes are not entirely the same. Consequently, the accuracy exhibited variations with different coal bases. The algorithm performs well when there is a high level of land cover consistency or when the coal base is relatively small. As the distance between the reference NDVI image and the to-be-calibrated NDVI image increases, the accuracy of the algorithm decreases. The accuracy with image distance >4 cannot be confirmed. While this study demonstrated the effectiveness of Auto-NDVI_cb_ at the coal-base scale, further research is needed at a larger spatial scale to explore the improvement of Auto-NDVI_cb_ in producing the NDVI across the whole of China and even the world.

Second, the accuracy of the annual maximum NDVI was closely related to the quality of the reference NDVI image. In an extreme case, the accuracy of the annual maximum NDVI may not be guaranteed when Landsat images are missing so severely that there is not an available image for the entire study area during periods of vigorous vegetation. Although such a case did not occur within the 14 coal bases examined in the past ten years, it remains a theoretical possibility when applying the algorithm to other regions.

## Data Availability

The GEE codes used to produce the annual maximum NDVI dataset is available to the public at https://code.earthengine.google.com/124ae202e3241a9523c0951ae6e4353f?noload=true.
